# The Edmonton Classification System for Cancer Pain in Patients with Bone Metastasis: a descriptive cohort study

**DOI:** 10.1007/s00520-023-07711-9

**Published:** 2023-04-28

**Authors:** Merlina Sulistio, Natalie Ling, Tara Finkelstein, Hoong Jiun Tee, Alexandra Gorelik, David Kissane, Natasha Michael

**Affiliations:** 1 Supportive, Psychosocial and Palliative Care Research Department, Cabrini Health, 181-183 Wattletree Rd, Mlavern, VIC 3144 Australia; 2 Faculty of Medicine, Nursing and Health Sciences, Monash University, Melbourne, VIC Australia; 3 School of Medicine, University of Notre Dame Australia Sydney, Sydney, NSW Australia; 4 Monash-Cabrini Department of Musculoskeletal Health and Clinical Epidemiology, Cabrini Health, Malvern, VIC Australia; 5 Department of Epidemiology and Preventive Medicine, School of Public Health and Preventive Medicine, Monash University, Melbourne, VIC Australia; 6 Department of Medicine (RMH), University of Melbourne, Melbourne, VIC Australia; 7 Sacred Heart Health Service, St. Vincent’s Hospital, Sydney, NSW Australia

**Keywords:** Pain control, Bone pain, Symptom assessment, Opioid

## Abstract

**Purpose:**

We describe the prevalence of the Edmonton Classification System for Cancer Pain (ECS-CP) features in patients with bone metastasis and cancer-induced bone pain (CIBP) and the relationship between ECS-CP features, pain intensity, and opioid consumption.

**Methods:**

We assessed ECS-CP features and recoded pain mechanisms and opioid use in adult patients with bone metastasis. Validated measures were used to assess pain intensity, incident pain, psychological distress, addictive behavior, and cognition.

**Results:**

Among 147 eligible patients, 95.2% completed the assessment. Mean participant age was 73.2 years, the majority female (52.1%) with breast cancer occurring most commonly (25.7%). One or more ECS-CP features were present in 96.4% and CIBP in 75.7% of patients. The median average and worst pain scores were 3 and 6, respectively. Neuropathic pain was the most prevalent pain mechanism (45.0%) and was associated with breakthrough pain frequency (*p*=0.014). Three-quarters had incident pain, which was strongly associated with a higher average and worst pain scores (3.5 and 7, *p*<0.001 for both), background oral morphine equivalent daily dose (26.7mg, *p*=0.005), and frequency of daily breakthrough analgesia (1.7 doses/day, *p*=0.007). Psychological distress (*n*=90, 64.3%) was associated with a significantly higher average pain score (4, *p*=0.009) and a slightly higher worst pain score (7, *p*=0.054). Addictive behaviour and cognitive dysfunction were relatively uncommon (18.6% and 12.9%, respectively).

**Conclusion:**

There is a need to promote standardized assessment and classification of pain syndromes such as CIBP. The ECS-CP may allow us to consider CIBP in a systematic manner and develop personalized pain interventions appropriate to the pain profile.

**Trial registration:**

Retrospectively registered in ANZCTR ACTRN12622000853741 (16/06/2022)

**Supplementary Information:**

The online version contains supplementary material available at 10.1007/s00520-023-07711-9.

## Introduction

Bone is a common site of metastasis and usually suggests non-curative cancer with a limited prognosis [1]. The majority (up to 80%) of bone metastasis arises from breast, prostate, and lung cancer [2, 3] and, in most cases, presents across multiple sites and involves the axial skeleton [4]. Significant morbidity and mortality are associated with bone metastasis due to complications such as hypercalcemia, pathological skeletal fractures, severe pain, impaired mobility, spinal cord compression, and bone marrow failure [1–3]. Bone metastasis results in particularly debilitating chronic pain [5], which traditionally presents with a continuous background pain, accompanied by episodes of the predictable incident and spontaneous breakthrough pain, significantly affecting the patient’s overall quality of life [6].

Cancer-induced bone pain (CIBP) is now recognized as a complex pain syndrome involving nociceptive, inflammatory, and neuropathic mechanisms [7]. The pathophysiology of pain in the setting of bone metastasis is related to the stimulation of inflammatory mediators and acidosis caused by bone-destructing osteoclasts via the increased expression of the receptor activator of nuclear factor k-B ligand (RANKL), resulting in mechanical destabilization and fractures [3, 8]. This, in turn, results in the sensitization and activation of mechanosensitive nerve fibers, the destruction of sensory nerve endings, and the pathological sprouting of sensory and sympathetic nerve fibers. The above contributes to peripheral and central sensitization of pain and the patient’s experience of chronic cancer pain [3, 9].

The prevalence of CIBP varies widely in the literature, with recent research confirming a prevalence of up to 92% [10]. A third of those with CIBP report severe pain, but clinical documentation of pain severity and the appropriate prescribing of analgesia remains unsatisfactory [10]. Up to a quarter of patients with CIBP suffer from neuropathic pain [11], and 40–75% report incident pain [11, 6], often of rapid onset (< 5 mins) and short duration (< 15 min) [6]. Incident pain, in particular, significantly affects daily life function [6] and causes psychological distress in up to 40% of patients [12]. The combined characteristics of CIBP, limited available appropriate therapies, and lack of standardized and routine screening contribute to ongoing reports of inadequately managed CIBP [8, 9].

ptimal pain management starts with systematic screening and identifying pain mechanisms [13]. The Edmonton Classification System for Cancer Pain (ECS-CP) is a standardized international classification system for cancer pain that integrates five features that predict pain management complexity: mechanism of pain, incident pain, psychological distress, addictive behaviour, and cognitive function [14, 15]. A multicenter international validation study of the ECS-CP investigated 1100 cancer patients and described a pain syndrome in 86% [14]. Younger patients (<60), those with neuropathic and incident pain, psychological distress, and higher pain intensity required a longer time to achieve stable pain control [14]. Additionally, independent of age, this cohort also required a higher mean oral morphine equivalent daily dose (OMEDD) and more co-analgesics to achieve stable pain control [14]. The median days to stable pain control increased as the number of prognostic factors (neuropathic pain, incident pain, psychological distress, age <60, and initial pain intensity) increased [16].  Further evaluation studies of the ECS-CP have identified neuropathic pain [17] and incident pain [11] as independent poor prognostic factors in cancer patients.

With the multidimensional nature of CIBP, a standardized application of the ECS-CP classification system may assist in identifying patients requiring more intensive pain management. This study aimed to evaluate the prevalence of the ECS-CP pain classification features in a cohort of patients with CIBP. The secondary aim was to examine the relationship between the ECS-CP features, pain intensity, daily opioid consumption, and breakthrough analgesic use. Ultimately, such a study may reveal the utility of the routine use of the ECS-CP in patient care.

## Method

### Study design and sample

This was a cross-sectional survey of consecutive cancer patients with bone metastasis conducted at an 850-bed metropolitan teaching hospital in Melbourne, Australia. Participants were recruited from the inpatient and ambulatory settings. Eligibility included: patients 18 years and older with a diagnosis of solid tumour or haematological malignancy and confirmation of bone metastasis on imaging. Patients who were unable to complete clinical assessment due to a language barrier, cognitive impairment, or deemed too unwell to participate as determined by treating clinicians were excluded. Ethics approval was obtained from the local research governance committee (No: 04-04-02-21), and completion of the survey as a component of routine care implied consent.

### Data and measures

A single patient assessment was conducted to collect all study data. The following demographic data were documented: age, sex, primary cancer diagnosis, and site of bone metastasis (categorized into long bones, spine, ribs, and/or pelvis). The following additional instruments were used:

## Edmonton Classification System for Cancer Pain (ECS-CP) [18]

The Edmonton Classification System for Cancer Pain (ECS-CP) (Appendix 1) is an international pain classification tool that evolved from the original instrument, the Edmonton Staging System, which was initially developed as a prognostic indicator for cancer pain management. It potentially identifies patients who may require complex pain management and provides a common language for pain classification to enable standardized reporting in research. The five discrete features of the ECS-CP allow for the assignment of a pain classification profile: mechanism of pain (N), incident pain (I), psychological distress (P), addictive behaviour (A), and cognitive function (C).

The presence of each discrete feature of the ECS-CP was determined as below:

### Mechanism of pain

The presence of nociceptive and/or neuropathic pain was determined at assessment by certified palliative care physicians through history taking, clinical examination, and correlation of findings with known sites of bone metastases. Neuropathic pain was deemed present if pain descriptors such as burning, electric shocks, shooting, pricking, tingling, pins and needles, or signs of hyper/hypoaesthesia were described by the patient or found on examination.

### Incident pain: Breakthrough Pain Assessment Tool (BAT) [19]

The BAT was developed and validated for the assessment of breakthrough cancer pain over the previous week and comprises 14 questions (9 relating to pain, 5 to pain treatment). Breakthrough cancer pain is defined as a transient pain exacerbation in patients with stable and controlled basal pain and may occur spontaneously or following predictable or unpredictable triggers [20. ]. We assessed the following components of the BAT: average daily frequency of incident pain, typical duration, and intensity of incident pain, and if any precipitating/relieving factors were present.

### Psychological distress: Distress Thermometer (DT) [21]

The DT is a validated, self-reported tool measuring patient distress over the previous week on a 0-to-10 rating scale (0, no distress; 10, extreme distress). Psychological distress was determined with a score ≥4 [22].

### Addictive behaviour: Cut down, Annoyed, Guilty and Eye-opener questionnaire adapted to include drugs (CAGE-AID) [23]

CAGE-AID is a validated screening tool used to detect drug and alcohol abuse, exploring four questions, with 1 point allocated for each positive response. We used a conservative cut-off point of ≥1 to suggest addictive behaviour. This has the sensitivity of detecting 91% of alcohol and 92% of drug abusers who are >50 years old [23].

### Cognitive impairment: Short Orientation Memory Concentration Test (SOMCT) [24]

The SOMCT is a 6-item memory and concentration test validated against neuropathology. It covers the assessment of orientation, concentration on a short task, and learning and recall of simple information. The patient scores 1 point for each incorrect answer, which is subsequently weighted (Appendix 2). The total score of SOMCT can discriminate between normal to minimal (0-–8), minimal-moderate (9–19), and severe cognitive (20–28) impairment. Patients with severe cognitive impairment were excluded from the study.

One point was allocated for each negative feature of the ECS-CP (presence of neuropathic pain, incident pain, psychological distress, addictive behaviour, and/or cognitive impairment) [25], which allowed for the calculation of the ECS-CP composite score ranging from 0 to 5 (total of all negative pain features) [26].

The following were also assessed:

## Pain intensity: 11-point numerical rating scale (NRS-11) [27]

Pain intensity in the previous 24 h was measured using the NRS-11 with 0 representing no pain, 1–3 mild pain, 3–4 moderate pain, and 7–10 severe pain. Patients were asked to rate their average and worst pain and the severity of their typical episode of breakthrough pain.

## Opioid requirements

The use of background and breakthrough opioid medications was determined from the inpatient electronic Medication Management charts or via direct participant reports or pain diaries for outpatients. The total dose of background analgesia (oral and parenteral) over the previous 24 h was calculated and converted to an OMEDD using established opioid conversion ratios [28]. The frequency of breakthrough opioid analgesia (BTA) use in 24 h was calculated by averaging the number of breakthrough doses over 72 h.

## Establishment of bone metastasis

The presence of bone metastasis was established by a review of radiological imaging and reports (plain film radiography, computed tomography, Technetium 99m bone scan, magnetic resonance imaging, and/or positron emission tomography) [4]. As CIBP most commonly arises from bone metastasis in the spine, pelvis, long bones, and ribs [2, 4, 29], only metastasis at these four sites was reported in this study.

### Statistical analysis

Patient demographics and pain characteristics were summarised using proportions for categorical variables, means and standard deviations (SDs) for normally distributed or medians, and inter-quartile ranges (IQRs) for continuous skewed data. The Mann-Whitney U test was used to examine the association between pain intensity, breakthrough pain characteristics, and opioid requirements, and the various ECS-CP features. Multivariable gamma regression analysis was used to analyze the relationship between ECSS-CP composite score and pain intensity while controlling for patients’ age and sex. A two-tailed *p* value <0.05 indicated statistical significance.

## Results

### Study participants

**Fig. 1:**
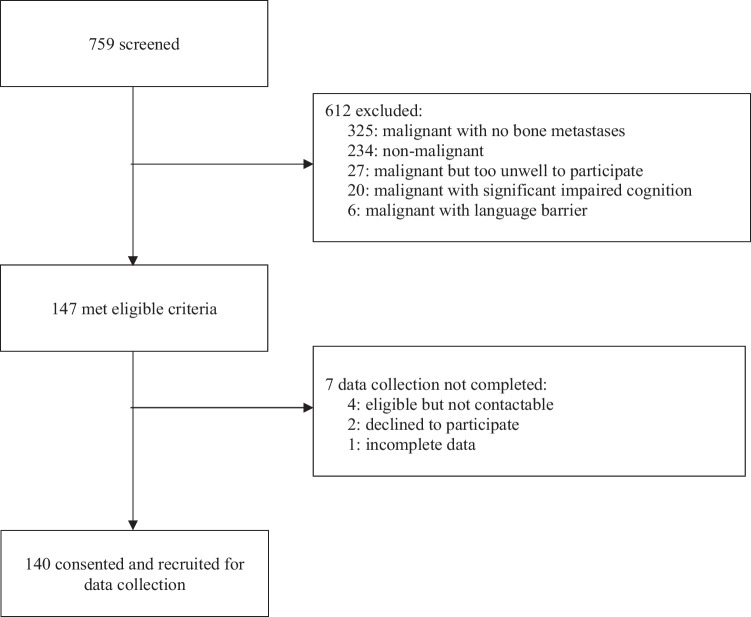
Subject selection

**Fig. 2:**
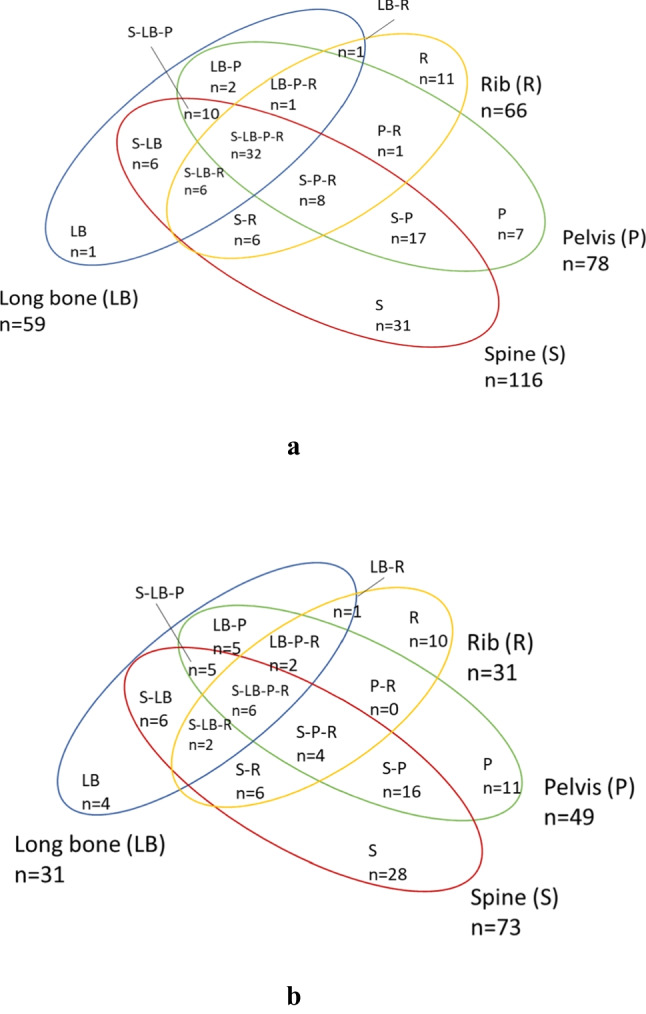
**a** Distribution of bone metastases in patients (*n*=140). **b** Distribution of *painful* bone metastases in patients (*n*=106)

Of the 147 eligible patients, 140 (95.2%) completed the survey (Fig. 1). Patient demographic and clinical characteristics are shown in Table 1. The mean (SD) age was 73.2 (11.2) years, with 47.9% male. The most common primary cancers were breast (25.7%), lung (24.3%), and prostate (20.0%). Bone metastasis was most commonly found in the spine (82.9%), with most patients (64.3%) having metastases across multiple sites. Thirty-two (22.9%) patients had bone metastases at all four sites, twenty-five (17.9%) at any three sites, and thirty-three (23.6%) patients at any two sites (Fig. 2). CIBP was present in 75.7% of patients, affecting approximately two-thirds of those with spinal or pelvic metastasis and approximately half of those with long bone and rib metastasis (Fig. 2).

**Table 1:** Patient demographics

**Patient characteristics (*n*=140)**	***n*** **(%)**
Age, mean in years (SD)	73.2 (11.2)
Male	67 (47.9)
Primary diagnosis	
Breast	36 (25.7)
Lung	34 (24.3)
Prostate	28 (20.0)
Gastrointestinal	14 (10)
Haematology	12 (8.6)
Other^a^	16 (11.4)
Site of bone metastases^b^	
Spine	116 (82.9)
Pelvis	78 (55.7)
Rib	66 (47.1)
Long bone	59 (42.1)

^a^ Genitourinary, other than prostate cancer (*n* =8), melanoma (*n* =3), carcinoma of unknown primary and other cancers (*n*=5)

^b^ The overall percentage is greater than 100% as multiple variables apply to some participants

### Pain and analgesia

Table 2 summarizes reported pain scores and analgesic use. The median reported average and worst pain intensity were 3 and 6, respectively. Moderate to severe (NRS ≥4) average and worst pain was reported by 42.8% and 67.6% of patients, respectively. Up to three-quarters of patients had been prescribed a background opioid. The median background opioid OMEDD was 25.8mg, and the median frequency of breakthrough opioid use was 1.3 doses/day. Oxycodone was the most commonly prescribed background (34.0%) and breakthrough (44.6%) opioid.

**Table 2:** Pain features and opioid use

**Pain intensity**	n (%)
Average pain (*n*=140), median (IQR)	3 (.75–5)
No pain (0)	34 (24.3)
Mild (1–3)	46 (32.9)
Moderate (4–6)	44 (31.4)
Severe (7+)	16 (11.4)
Worst pain (*n*=139), median (IQR)	6 (2–8)
No pain (0)	27 (19.4)
Mild (1–3)	18 (13.0)
Moderate (4–6)	27 (19.4)
Severe (7+)	67 (48.2)
**Pain mechanism**	
Nociceptive	51 (36.4)
Neuropathic with/without nociceptive	63 (45.0)
No pain syndrome	24 (17.1)
Insufficient data to classify	2 (1.4)
**Incident pain**	**104 (74.3)**
Frequency	
Less than once a day	15 (14.4)
1–2 times a day	27 (26.0)
3–4 times a day	33 (31.7)
>4 times a day	29 (27.9)
Duration	
<5 min	24 (23.1)
5–15min	31 (29.8)
15–30min	20 (19.2)
30–60min	12 (11.5)
>60min	15 (14.4)
Missing data	2 (1.9)
Typical pan intensity (*n*=70), median (IQR)	6 (4.25–8)
**Psychological distress**	**90 (64.3)**
Distress thermometer (median, IQR)	5 (2–7)
**Addictive behaviour**	**26 (18.6)**
CAGE-AID score (median, IQR)	0 (0)
**Cognitive impairment**	**18 (12.9)**
SOMCT (median, IQR)	4 (0–6)
**Opioid Use**	
*Background opioid (n=103)*	
Oxycodone	35/103 (34.0)
Fentanyl	20/103 (19.4)
Methadone	16/103 (15.5)
Morphine	15/103 (14.6)
Hydromorphone	9/103 (8.7)
Others	8/103 (7.8)
OMEDD, median in mg (IQR)	25.8 (6.7–47)
*Breakthrough opioid*^*a*^ *(n=101)*	
Oxycodone	45/101 (44.6)
Fentanyl	33 /101 (32.7)
Morphine	24/101 (23.8)
Hydromorphone	18/101 (17.8)
Others	2/101 (2.0)
Daily frequency, median number of doses (IQR)	1.3 (0.3–2.7)

*CAGE-AID*, Cut down, annoyed, guilty, and eye-opener-adapted to include drugs; *ECS-CP*, Edmonton classification system for cancer pain; *IQR*, Interquartile range; *OMEDD*, oral morphine equivalent daily dose; *SOMCT*, Short orientation memory concentration test

^a^ The overall percentage is greater than 100% as multiple variables apply to some participants

### Edmonton Classification System for Cancer Pain Features

One or more ECS-CP features were present in 135 patients (Table 2). *Neuropathic pain* was reported in 45% of patients [with the source of painful metastasis arising from the spine (47), rib and pelvis (6 each), and long bones (4)]. Neuropathic pain was associated with a higher reported frequency of breakthroughs (p=0.014). Although it was the most prevalent mechanism of pain reported, it did not impact pain intensity or opioid requirements (Table 3). Three-quarters of patients reported having *incident pain*, with a reported median incident pain score of 6. Over half of the patients reported a daily average of ≥ three episodes of incident pain; for 52.9% of patients, these episodes lasted ≤ 15 min. Of the ninety-one patients who identified triggers to their incident pain, the majority of patients had predictable pain with movement. The presence of incident pain was strongly associated with higher average and worst pain scores (*p*<0.001 for both), higher background OMEDD (*p*=0.005), and a higher frequency of daily BTA use (*p*=0.007) (Table 2).

**Table 3:** Association between the Edmonton Classification System for Cancer Pain features, pain intensity and Opioid requirement*

ECSCP features		Average pain	Worst pain	Background OMEDD^#^, mg	Breakthrough opioid frequency^#^
Pain mechanism	Nociceptive Neuropathic	3 (2–5) 4 (2–5) *P*=0.510	6.5 (3–8) 7 (5–9) *P* =0.105	25 (6.7–47) 26.7 (4.8–60) *P* =0.539	1.5 (1–3) 1.3 (0.3–2.8) *P* =0.746
Incident pain	Present Absent	3.5 (2–5) 0 (0–2) *P* <0.001	7 (5–8) 0 (0–4) *P* <0.001	26.7 (6.7–60.0) 11.7 (6.7–18.0) *P* =0.005	1.7 (0.7–3.0) 0.7 (0.3–1.3) *P* =0.007
Psychological distress	Present Absent	4 (0.5–5) 2 (1–3) *P* =0.009	7 (2–8) 5 (2–7) *P* =0.054	26.7 (6.7–60.0) 23.5 (1.2–30.0) *P* =0.238	1.2 (0.3–2.8) 1.3 (0.7–2.8) *P* =0.988
Addictive behaviour	Present Absent	4 (2–5) 2 (0–4) *P* =0.099	6.5 (5–8) 6 (2–8) *P* =0.497	33.8 (7.0–60) 21.8 (6.7–40.0) *P* =0.268	2.0 (0.3–3.3) 1.0 (0.3–2.3) *P* =0.217
Cognitive dysfunction	Present Absent	4 (0–6) 3 (0.75–4) *P* =0.298	7 (0–8) 6 (2–8) *P* =0.825	30 (18–60) 24 (3.2–47) *P* =0.221	1.0 (0.7–1.7) 1.3 (0.3–3) *P* =0.409

*OMEDD*, Oral Morphine Equivalent Daily Dose

*Mann-Whitney U-test; ^#^values are based on those who had opioids

Results reported as median (IQR)

Two-thirds of patients reported *psychological distress*, associated with significantly higher average pain scores (p=0.009) and slightly higher worst pain scores (p=0.054). However, no correlation with opioid requirements was found (Tables 2 and 3). *Addictive behaviour* and *cognitive dysfunction* were relatively uncommon (18.6% and 12.9%, respectively) and were not associated with pain intensity or opioid requirements.

**Fig. 3:**
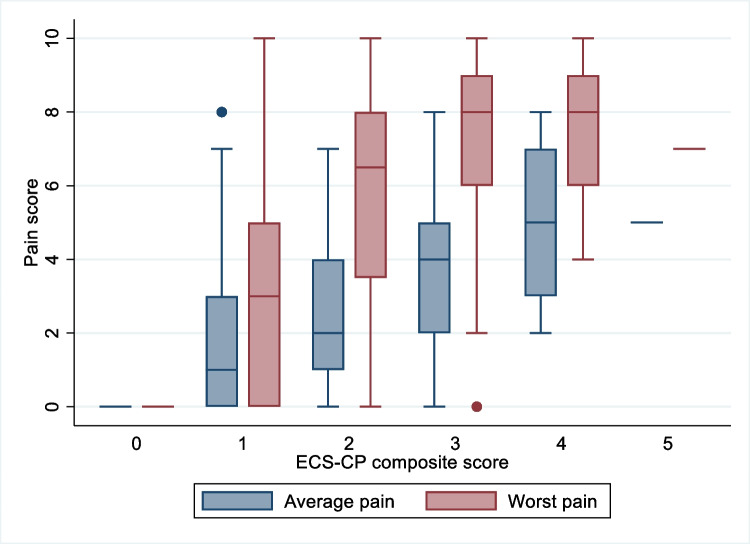
Association of ECS-CP composite score and pain intensity. ECS-CP, Edmonton classification system for cancer pain. ECS-CP composite score 0 to 5; *n* (%), respectively: 5 (3.6), 37 (26.4), 44 (31.4), 38 (27.1), 15 (10.7), 1 (0.7).

The association between pain and *ECS-CP composite score* is depicted in Fig. 3. Higher average and worst pain were associated with higher ECS-CP composite scores (*p*<0.001 for both). This association remained significant after adjustment for a patient’s age and gender, with a 1-point increase in the ECS-CP composite score leading to 43.6% (95% CI 22.6–68.2) and 46.9% (95% CI 28.5–67.9) increases in average and worst pain respectively.

## Discussion

This is the first study to systematically explore the ECS-CP features in a cohort of adult cancer patients with bone metastasis and CIBP. We confirm the previously reported high prevalence of neuropathic pain, incident pain, and psychological distress in cancer patients with bone metastases, with spinal metastasis being the most common site for CIBP. We detected a strong association between incident pain, psychological distress, and pain intensity, with incident pain being the only factor associated with higher opioid requirements (background OMEDD and daily BTA frequency). Finally, we describe sites of metastasis, with the spine as the most common site, and correlated reports of CIBP at these sites as previously reported [29].

Current treatment for CIBP can be classified into anticancer treatments (local radiotherapy, radioisotopes and systemic chemotherapeutics, and immunotherapy), analgesic treatment using opioids, anti-inflammatory drugs, and co-analgesics (antidepressants, anticonvulsants, and gabapanetinoids), bone-targeted therapies (such as bisphosphonate and denosumab) and surgery [3, 8]. Local radiotherapy, a commonly used modality has a reported pain response rate of 61% with a median response time of 4 weeks [30]. Whilst there has been increased interest in the study of bone pain [31], a translational paradigm has not successfully allowed the pathophysiology and mechanism of CIBP to inform the choice of pharmacotherapeutics [32]. Opioids remain the mainstay treatment with no evidence to guide the choice of the opioid molecule [3]. More recently, a multicenter randomized controlled study of 223 patients with CIBP receiving radiotherapy was allocated to receive pregabalin (targeting the neuropathic element of CIBP) or placebo. The study findings did not support the role of pregabalin in CIBP, with no significant difference in average pain or pain interference compared to placebo [33].

In recognition of the ongoing challenges of managing complex cancer pain, recommendations have been made for developing and implementing a widely recognized and standardized taxonomy and classification system for cancer pain [34]. More recently, the International Classification of Disease–11 was updated to reflect a new classification system for chronic cancer pain to aid the development of individualized management plans and stimulate research in pain syndromes [35. ]. Chronic cancer pain is now subdivided in this new classification system into four categories: visceral, bone, neuropathic, and “other,” such as chronic bone cancer pain and chronic neuropathic pain.

Thus far, the ECS-CP remains the most validated classification system for cancer pain [18]. The ECS-CP highlights the multidimensional nature of pain assessment and classification, delineating distinct and independent categories that can influence pain management outcomes. When compared to published data on ECS-CP features in cancer patients, our cohort with bone metastases reported a higher prevalence of neuropathic pain features (45% vs 16.9–35%) [11, 14, 36, 26, 17], experienced more incident pain (74.3% vs 28–61%) [11, 37, 14, 36, 26, 17], psychological distress (64.3% vs 25–52.7%) [14, 36, 26, 17], and addictive behaviour (18.6% vs 4–11%) [14, 36, 26, 17], but reported a similar prevalence of cognitive dysfunction (12.9% vs 3–21%) [14, 36, 26, 17]. We also reported a similar average (3 vs 4) and worst (6 vs 7) pain scores to that found in a regional European oncology cohort of fifty-five patients with bone metastasis [6]. Despite confirming the influence of incident pain on average and worst pain intensity, we did not observe a relationship between neuropathic pain, pain intensity, and OMEDD use as reported in other studies [14, 26, 17]. Finally, we demonstrated a correlation between the ECS-CP composite scores and pain intensity.

A retrospective study of 386 American cancer patients showed that those with neuropathic pain were less likely to achieve their pain goals [17]. This is unsurprising as neuropathic pain remains a significant unmet medical need, requiring a multimodal approach to care [38]. It is challenging to ascertain clearly in our study if the neuropathic element reported arose specifically from CIBP, commonly described as mixed cancer pain or cancer treatments [39, 40], as there are no clinical guidelines as to how to best clinically assess the neuropathic element in CIBP. Nonetheless, its identification through routine screening is critical for considering appropriate pharmacotherapeutics [41] and other modalities due to the strong known association between poorly controlled neuropathic pain and overall quality of life [42].

Likewise, a multicenter international study of 606 patients with bone metastases confirmed the relationship between incident pain and worst pain intensity and its negative prognostic value at a month follow-up [11]. Incident pain is a subtype of breakthrough pain that occurs with normal voluntary (e.g. walking) or involuntary (e.g. cough) movement but is typically absent at rest [37]. Our findings with participants reporting an average of 3 breakthrough episodes a day, commonly lasting 5–15 min, mirror that of a European study of the characteristics of breakthrough cancer pain in 1000 oncology patients [37]. However, an ongoing challenge in clinical practice is the lack of congruence between the temporal pattern of the predictable incident pain that commonly occurs in CIBP (rapid onset and short duration) and the pharmacokinetics of the immediate-release opioids currently prescribed. Commonly used immediate-release formulations of oxycodone, morphine and hydromorphone (Table 2) provide a delayed onset of analgesia compared to the onset of incident pain. Thus, the onset of analgesia occurs after the episode of pain subsides, and the effects of analgesia last beyond the episode of pain. This, in turn, leads to patients commonly complaining of opioid-induced adverse effects. Thus, formulations of opioids with a rapid onset of analgesia and short duration of action, mimicking the temporal pattern of predictable incident pain in CIBP, may be the more appropriate opioid of choice [43].

This study has several limitations, being a single-site study of cancer patients with CIBP. We did not record data on non-opioid analgesics/ co-analgesics which may have affected overall pain scores and may explain the cohort of patients who report no CIBP. We only reported pain from the four most common sites of bone metastases. Furthermore, the cross-sectional study design limits the evaluation of the prognostic capabilities of the different ECS-CP features in guiding pain management. Using a standardized tool to screen for neuropathic pain, such as the Leeds Assessment of Neuropathic Symptom and Signs [44] or the Douleur Neuropathique 4 [45], seeking to further identify potential aetiologies of pain and applying the neuropathic pain grading system of possible, probable and definite neuropathic pain [46], may have improved our reporting and assessment of neuropathic pain. Finally, our ECS-CP composite score requires interpretation with caution as the number of patients with pain scores ≥4 remains small.

## Clinical implications

Considering the complexity of the pathophysiology of CIBP, the ECS-CP may allow us to consider CIBP more systematically and thus develop personalized pain management interventions according to the pain profile identified. It further allows for targeting more specialized input for patients with a high prevalence of neuropathic or incident pain and psychological distress. Targeted intervention studies may further demonstrate the utility of the ECS-CP in the clinical setting.

## Conclusion

There is a need to promote a more standardized way to assess and classify pain syndromes such as CIBP. Our approach requires consideration of the multifactorial aetiology of CIBP that includes nociceptive, inflammatory, and neuropathic components and warrants various modalities to manage symptoms in conjunction with disease-modifying therapies. Epidemiological, clinical, and translational data may provide avenues for us to consider how we further target and manage CIBP for each individual that experiences it.

## Supplementary information

ESM 1

ESM 2

## Data Availability

Cabrini Research Institute retains primary control of the data presented in this manuscript. Data may be made available for external review if permission is obtained from the Cabrini Research Institute.

## References

[1] Macedo F, Ladeira K, Pinho F, Saraiva N, Bonito N, Pinto L, Goncalves F (2017). Bone Metastases: An Overview Oncol Rev.

[2] Coleman RE (2006). Clinical features of metastatic bone disease and risk of skeletal morbidity. Clin Cancer Res.

[3] Zajaczkowska R, Kocot-Kepska M, Leppert W, Wordliczek J (2019) Bone pain in cancer patients: mechanisms and current treatment. Int J Mol Sci 20(23). 10.3390/ijms20236047 10.3390/ijms20236047 PMC6928918 31801267

[4] O’Sullivan GJ, Carty FL, Cronin CG (2015). Imaging of bone metastasis: an update. World J Radiol.

[5] Ahmad I, Ahmed MM, Ahsraf MF, Naeem A, Tasleem A, Ahmed M, Farooqi MS (2018). Pain management in metastatic bone disease: a literature review. Cureus.

[6] Laird BJ, Walley J, Murray GD, Clausen E, Colvin LA, Fallon MT (2011). Characterization of cancer-induced bone pain: an exploratory study. Support Care Cancer.

[7] Falk S, Dickenson AH (2014). Pain and nociception: mechanisms of cancer-induced bone pain. J Clin Oncol.

[8] Middlemiss T, Laird BJ, Fallon MT (2011). Mechanisms of cancer-induced bone pain. Clin Oncol (R Coll Radiol).

[9] Mantyh PW (2014). Bone cancer pain: from mechanism to therapy. Curr Opin Support Palliat Care.

[10] Vieira C, Fragoso M, Pereira D, Medeiros R (2019). Pain prevalence and treatment in patients with metastatic bone disease. Oncol Lett.

[11] Habberstad R, Hjermstad MJ, Brunelli C, Kaasa S, Bennett MI, Pardon K, Klepstad P (2019). Which factors can aid clinicians to identify a risk of pain during the following month in patients with bone metastases?. A longitudinal analyses Support Care Cancer.

[12] Canal-Sotelo J, Trujillano-Cabello J, Larkin P, Arraras-Torrelles N, Gonzalez-Rubio R, Rocaspana-Garcia M, Barallat-Gimeno E (2018). Prevalence and characteristics of breakthrough cancer pain in an outpatient clinic in a Catalan teaching hospital: incorporation of the Edmonton Classification System for Cancer pain into the diagnostic algorithm. BMC Palliat Care.

[13] Caraceni A, Shkodra M (2019) Cancer pain assessment and classification. Cancers (Basel) 11(4). 10.3390/cancers11040510 10.3390/cancers11040510 PMC6521068 30974857

[14] Fainsinger RL, Nekolaichuk C, Lawlor P, Hagen N, Bercovitch M, Fisch M, Galloway L, Kaye G, Landman W, Spruyt O, Zhukovsky D, Bruera E, Hanson J (2010). An international multicentre validation study of a pain classification system for cancer patients. Eur J Cancer.

[15] Nekolaichuk CL, Fainsinger RL, Lawlor PG (2005). A validation study of a pain classification system for advanced cancer patients using content experts: the Edmonton Classification System for Cancer Pain. Palliat Med.

[16] Nekolaichuk C, Fainsinger RL, Lawlor P, Hagen N, Berkovitch M. Fisch M, Galloway L, Kaye G, Landman W, Spruyt O, Zhukovsky D, Bruera E, Hanson J. (2010) A predictive model for identifying complex cancer pain syndromes in patients referred to specialist palliative care services using a pain classification system [abstract]. Palliat Med 24(4, Suppl):S42

[17] Arthur J, Tanco K, Haider A, Maligi C, Park M, Liu D, Bruera E (2017). Assessing the prognostic features of a pain classification system in advanced cancer patients. Support Care Cancer.

[18] Lawlor PG, Lawlor NA, Reis-Pina P (2018). The Edmonton Classification System for Cancer Pain: a tool with potential for an evolving role in cancer pain assessment and management. Expert Rev Qual Life in Cancer Care.

[19] Webber K, Davies AN, Zeppetella G, Cowie MR (2014). Development and validation of the breakthrough pain assessment tool (BAT) in cancer patients. J Pain Symptom Manag.

[20] Davies AN, Dickman A, Reid C, Stevens AM, Zeppetella G, Science Committee of the Association for Palliative Medicine of Great B, Ireland (2009). The management of cancer-related breakthrough pain: recommendations of a task group of the Science Committee of the Association for Palliative Medicine of Great Britain and Ireland. Eur J Pain.

[21] Ownby KK (2019). Use of the distress thermometer in clinical practice. J Adv Pract Oncol.

[22] Donovan KA, Grassi L, McGinty HL, Jacobsen PB (2014). Validation of the distress thermometer worldwide: state of the science. Psychooncology.

[23] Hinkin CH, Castellon SA, Dickson-Fuhrman E, Daum G, Jaffe J, Jarvik L (2001). Screening for drug and alcohol abuse among older adults using a modified version of the CAGE. Am J Addict.

[24] Wade DT, Vergis E (1999). The Short Orientation-Memory-Concentration Test: a study of its reliability and validity. Clin Rehabil.

[25] Bruera E, Schoeller T, Wenk R, MacEachern T, Marcelino S, Hanson J, Suarez-Almazor M (1995). A prospective multicenter assessment of the Edmonton staging system for cancer pain. J Pain Symptom Manag.

[26] Arthur J, Yennurajalingam S, Nguyen L, Tanco K, Chisholm G, Hui D, Bruera E (2015). The routine use of the Edmonton Classification System for Cancer Pain in an outpatient supportive care center. Palliat Support Care.

[27] Atisook R, Euasobhon P, Saengsanon A, Jensen MP (2021). Validity and utility of four pain intensity measures for use in international research. J Pain Res.

[28] Caraceni A, Hanks G, Kaasa S, Bennett MI, Brunelli C, Cherny N, Dale O, De Conno F, Fallon M, Hanna M, Haugen DF, Juhl G, King S, Klepstad P, Laugsand EA, Maltoni M, Mercadante S, Nabal M, Pigni A, Radbruch L, Reid C, Sjogren P, Stone PC, Tassinari D, Zeppetella G, European Palliative Care Research C, European Association for Palliative C (2012). Use of opioid analgesics in the treatment of cancer pain: evidence-based recommendations from the EAPC. The Lancet Oncol.

[29] Kakhki VR, Anvari K, Sadeghi R, Mahmoudian AS, Torabian-Kakhki M (2013). Pattern and distribution of bone metastases in common malignant tumors. Nucl Med Rev Cent East Eur.

[30] van der Velden JM, van der Linden YM, Versteeg AL, Verlaan JJ, Sophie Gerlich A, Pielkenrood BJ, Kasperts N, Verkooijen HM (2018). Evaluation of effectiveness of palliative radiotherapy for bone metastases: a prospective cohort study. J Radiat Oncol.

[31] Zhen G, Fu Y, Zhang C, Ford NC, Wu X, Wu Q, Yan D, Chen X, Cao X, Guan Y (2022). Mechanisms of bone pain: progress in research from bench to bedside. Bone Res.

[32] Park SH, Eber MR, Widner DB, Shiozawa Y (2018). Role of the bone microenvironment in the development of painful complications of skeletal metastases. Cancers (Basel).

[33] Fallon M, Hoskin PJ, Colvin LA, Fleetwood-Walker SM, Adamson D, Byrne A, Murray GD, Laird BJ (2016). Randomized double-blind trial of pregabalin versus placebo in conjunction with palliative radiotherapy for cancer-induced bone pain. J Clin Oncol.

[34] Bennett MI (2010). Cancer pain terminology: time to develop a taxonomy that promotes good clinical practice and allows research to progress. Pain.

[35] Bennett MI, Kaasa S, Barke A, Korwisi B, Rief W, Treede RD, Pain ITftCoC (2019). The IASP classification of chronic pain for ICD-11: chronic cancer-related pain. Pain.

[36] Nekolaichuk CL, Fainsinger RL, Aass N, Hjermstad MJ, Knudsen AK, Klepstad P, Currow DC, Kaasa S, European Palliative Care Research C (2013). The Edmonton Classification System for Cancer Pain: comparison of pain classification features and pain intensity across diverse palliative care settings in eight countries. J Palliat Med.

[37] Davies A, Buchanan A, Zeppetella G, Porta-Sales J, Likar R, Weismayr W, Slama O, Korhonen T, Filbet M, Poulain P, Mystakidou K, Ardavanis A, O’Brien T, Wilkinson P, Caraceni A, Zucco F, Zuurmond W, Andersen S, Damkier A, Vejlgaard T, Nauck F, Radbruch L, Sjolund KF, Stenberg M (2013). Breakthrough cancer pain: an observational study of 1000 European oncology patients. J Pain Symptom Manag.

[38] Davis MP (2018). Cancer-related neuropathic pain: review and selective topics. Hematol Oncol Clin North Am.

[39] Lee DY, Lee JJ, Richeimer SH (2021). Cancer pain syndromes. Cancer Treat Res.

[40] Fallon MT (2013) Neuropathic pain in cancer. Br J Anaesth 111 (1):105-111. 10.1093/bja/aet208 10.1093/bja/aet208 23794652

[41] Finnerup NB, Attal N, Haroutounian S, McNicol E, Baron R, Dworkin RH, Gilron I, Haanpaa M, Hansson P, Jensen TS, Kamerman PR, Lund K, Moore A, Raja SN, Rice AS, Rowbotham M, Sena E, Siddall P, Smith BH, Wallace M (2015). Pharmacotherapy for neuropathic pain in adults: a systematic review and meta-analysis. Lancet Neurol.

[42] Ulas S, Eyigor S, Caramat I (2018). Quality of life and neuropathic pain in hospitalized cancer patients: a comparative analysis of patients in palliative care wards versus those in general wards. Indian J Palliat Care.

[43] Brzakala J, Leppert W (2019). The role of rapid onset fentanyl products in the management of breakthrough pain in cancer patients. Pharmacol Rep.

[44] Bennett M (2001). The LANSS Pain Scale: the Leeds assessment of neuropathic symptoms and signs. Pain.

[45] Bouhassira D, Attal N, Alchaar H, Boureau F, Brochet B, Bruxelle J, Cunin G, Fermanian J, Ginies P, Grun-Overdyking A, Jafari-Schluep H, Lanteri-Minet M, Laurent B, Mick G, Serrie A, Valade D, Vicaut E (2005). Comparison of pain syndromes associated with nervous or somatic lesions and development of a new neuropathic pain diagnostic questionnaire (DN4). Pain.

[46] Finnerup NB, Haroutounian S, Kamerman P, Baron R, Bennett DLH, Bouhassira D, Cruccu G, Freeman R, Hansson P, Nurmikko T, Raja SN, Rice ASC, Serra J, Smith BH, Treede RD, Jensen TS (2016). Neuropathic pain: an updated grading system for research and clinical practice. Pain.

