# Supplementary material for: The Edmonton Classification System for Cancer Pain in Patients with Bone Metastasis: a descriptive cohort study
Source: Support Care Cancer. 2023 Apr 28;31(5):305. doi: 10.1007/s00520-023-07711-9 (PMC10140090; doi:10.1007/s00520-023-07711-9)
Supplement: Supplementary file 2 — ESM 2 [file 520_2023_7711_MOESM2_ESM.docx]

**Appendix 2 Short Orientation Memory Concentration Test**
